# Unilateral facial edema after filler injection of the lower eyelid

**DOI:** 10.1111/dth.13539

**Published:** 2020-06-30

**Authors:** Tom S. Decates, Elmer C. Kruijt Spanjer, Renu Saini, Peter J. Velthuis, Frank M. Niessen

**Affiliations:** ^1^ Department of Dermatology Erasmus Medical Center Rotterdam Netherlands; ^2^ Department of Maxillo and Facial Surgery Haaglanden Medisch Centrum the Hague Netherlands; ^3^ Meyer Dental Practice the Hague Netherlands; ^4^ Department of Plastic Surgery Amsterdam University Medical Center Amsterdam Netherlands

**Keywords:** cosmetic dermatology, dermal, edema, eyelid, facial, filler, fillers, surgery, unilateral

## Abstract

The use of hyaluronic acid (HA) gel fillers for rejuvenation of the face has been increasing in popularity over the years. This nonsurgical, temporary technique is commonly used in the periocular region to restore volume. The aim of this study was creating awareness in the potential causes of edema after hyaluronic acid gel filler injections under the eyes. A 32‐year‐old woman presented for a cosmetic consultation to address unilateral swelling of the left check. She states she had an HA filler injected in the tear trough on both sides. Extensive evaluation and ultrasound were performed by physicians of different specialties. Intra‐oral and radiological examination revealed a tooth‐related cause known as apical periodontitis. Removal of this tooth resulted in complete resolution of the patient's presenting symptoms. Familiarity with all the potential causes of adverse events after injections with hyaluronic acid gel fillers accelerates the treatment and healing of the patient with complications. Reporting this case should raise awareness about possible teeth‐related complications.

## 1 INTRODUCTION

The use of hyaluronic acid gel fillers for rejuvenation of the face has been increasing in popularity over the years.^1^, ^2^ This nonsurgical technique with a temporary effect is commonly used in the periocular region to restore volume. Unfortunately, in 11% of the treatments, this so‐called “tear trough” with hyaluronic acid gel filler malar edema occurs.^3^ In this report, we present an unusual case of unilateral edema, which occurred 2 weeks after placement of filler in the tear trough on both sides.

## 2 CASE REPORT

**FIGURE 1:**
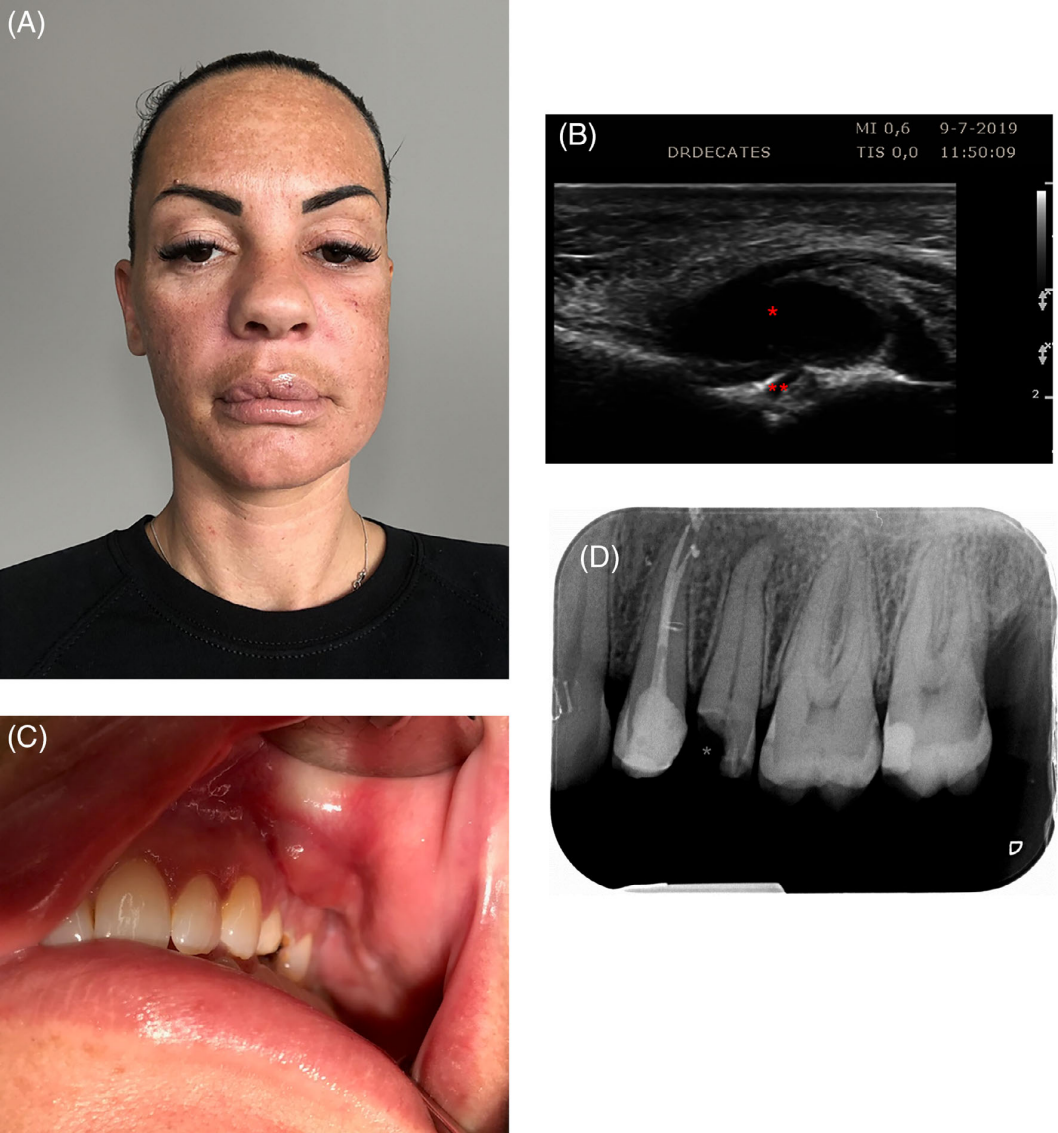
A, Appearance of the edema of the left cheek at the initial visit. B, Ultrasound image of the left cheek clearly showing a hypo‐echogenic area, suspect for abscess and possibly a fistula. C, An abscess and decayed second bicuspid in the left side of the upper jaw. D, Radiological examination of tooth nr 25 showing deep caries with a decayed part of the crown

A 32‐year‐old woman presented for a cosmetic consultation to address unilateral swelling of the left cheek (Figure 1A). She states she had a hyaluronic acid filler injected (Stylage S, 0,5 mL per side) in the tear trough on both sides, with a canula, 2 weeks earlier. Ten days after the treatment, she began to notice swelling of the area underneath her left eye, slowly expending toward the left cheek. The original injector already injected 50 units of hyaluronidase into the left tear trough, without any result. Her medical history shows a purified medical silicone (PMS) treatment of the lips 10 years earlier. Physical examination revealed unilateral orbital and cheek edema with a palpable large mass in the left cheek. Ultrasound is very helpful in cases of adverse events after being injected with hyaluronic acid gel fillers.^4^ With this a large hypo‐echogenic area, interpreted as an abscess, was visible and there existed a hypo‐echogenic tract toward the second bicuspid on the left side of the maxilla (tooth nr 25), possibly a fistula (Figure 1B). A closer look inside the mouth showed a decayed second bicuspid in the left side of the upper jaw and she was therefore referred to the dentist (Figure 1C). Consultation by the dentist revealed deep caries of tooth 25 with a painful swelling of the buccal corridor. Radiological examination showed an apical radiolucency, highly suggestive of apical periodontitis (Figure 1D). The patient was then seen by the oral and maxillofacial surgeon. Extraction of the tooth from its socket was followed by drainage of a purulent fluid. The patient felt instant relieve of the pressure she felt before the treatment. After 1 week, the edema fully disappeared.

## 3 DISCUSSION

The exact etiology of the edema after filler injections is poorly understood.^3^ The edema extends outside the borders of the injection site and may represent a low‐grade inflammatory reaction; however, there is usually an absence of any hard signs of inflammation.^3^ DeLorenzi suggests that some patients may have, or develop, a hypersensitivity to the HA and thus, the noninfective edema may be a result of this.^5^ A PubMed search was conducted to identify articles about unilateral edema after dermal filler injections, but none was found. Thus, when a patient visits a clinic with unilateral edema, one should also consider other possible causes.

Tooth decay is a common problem in the modern world. If left untreated, all infected root canals will lead to apical periodontitis. The golden standard to diagnose apical periodontitis is the combination of intraoral examination and two‐ or three‐dimensional radiological examination. An ultrasound can also be used in the case of soft tissue swelling with a sinus tract or fistula.^6^ Long‐standing apical periodontitis can lead to chronic elevation of inflammatory response.^7^ Treatment comprises a root canal treatment or removal of the affected tooth. Both options lead to removal of the infection and drainage of any abscess formation.

Familiarity with all the possible causes of adverse events after injections with hyaluronic acid gel fillers accelerates the treated and healing of the patient with complications. Reporting this case should raise awareness about possible teeth‐related complications.

## CONSENT

Written informed consent was obtained from the patient for publication of this case report. A copy of the written consent is available for review by the editor of this journal.

## CONFLICT OF INTEREST

The authors declare no conflict of interest.
